# Inverse tissue mechanics of cell monolayer expansion

**DOI:** 10.1371/journal.pcbi.1006029

**Published:** 2018-03-01

**Authors:** Yohei Kondo, Kazuhiro Aoki, Shin Ishii

**Affiliations:** 1 Graduate School of Informatics, Kyoto University, Kyoto, Japan; 2 Division of Quantitative Biology, National Institute for Basic Biology, Aichi, Japan; 3 Department of Basic Biology, Faculty of Life Science, SOKENDAI (Graduate University for Advanced Studies), Aichi, Japan; Oxford, UNITED KINGDOM

## Abstract

Living tissues undergo deformation during morphogenesis. In this process, cells generate mechanical forces that drive the coordinated cell motion and shape changes. Recent advances in experimental and theoretical techniques have enabled *in situ* measurement of the mechanical forces, but the characterization of mechanical properties that determine how these forces quantitatively affect tissue deformation remains challenging, and this represents a major obstacle for the complete understanding of morphogenesis. Here, we proposed a non-invasive reverse-engineering approach for the estimation of the mechanical properties, by combining tissue mechanics modeling and statistical machine learning. Our strategy is to model the tissue as a continuum mechanical system and to use passive observations of spontaneous tissue deformation and force fields to statistically estimate the model parameters. This method was applied to the analysis of the collective migration of Madin-Darby canine kidney cells, and the tissue flow and force were simultaneously observed by the phase contrast imaging and traction force microscopy. We found that our monolayer elastic model, whose elastic moduli were reverse-engineered, enabled a long-term forecast of the traction force fields when given the tissue flow fields, indicating that the elasticity contributes to the evolution of the tissue stress. Furthermore, we investigated the tissues in which myosin was inhibited by blebbistatin treatment, and observed a several-fold reduction in the elastic moduli. The obtained results validate our framework, which paves the way to the estimation of mechanical properties of living tissues during morphogenesis.

## Author summary

In order to shape the body of a multicellular organism, cells generate mechanical forces and undergo deformation. Although these forces are being increasingly determined, quantitative characterization of the relation between the deformation and forces at the tissue level remains challenging. To estimate these properties, we developed a reverse-engineering method by combining tissue mechanics modeling and statistical machine learning, and then tested this method on a common model system, the expansion of cultured cell monolayer. This statistically sound framework uses the passive observations of spontaneous deformation and force dynamics in tissues, and enables us to elucidate unperturbed mechanical processes underlying morphogenesis.

## Introduction

The body of multicellular organisms must be properly shaped in order to exert its functions, and this proper formation is based on the orchestration of cellular behaviors, such as cell division, differentiation, migration, and other. One of the key processes in morphogenesis is the coordinated change in cell shapes and positions. The coordination depends on cell-generated mechanical forces that introduce stress, which induces multicellular deformation and flow [1]. Therefore, the research on how the molecular components responsible for force generation and propagation, such as motor proteins and cell-cell adhesion molecules, are regulated in space and time during the morphogenesis has attracted a lot of attention recently [2]. In parallel, remarkable progress has been made in the development of the technologies allowing the measurements of the generated forces and stress in the living tissues [3], which represents a crucial step towards linking the underlying molecular activities with the morphogenesis.

Epithelial tissues represent important model systems for the understanding of the force dynamics during morphogenesis, because their two-dimensional sheet structure facilitates the observation of the processes that occur in these tissues and analysis. In particular, many valuable insights have been obtained using the cultured cell monolayer, i.e., one-cell-thick sheet of tightly-connected epithelial cells [4–6]. The cells belonging to a monolayer collectively migrate in order to fill a cell-free surface, which replicates *in vivo* tissue remodeling, such as wound repair, which occurs during regeneration, and epiboly, during embryonic development. When migrating, the cells exert forces on the underlying substrate to propel themselves forward, and in the unicellular motion, this force, known as the cell traction force, can be visualized by the displacement of fluorescent beads embedded into the substrate [7]. The simple flat-sheet structure of the monolayer allows us to apply the same technique to observe a spatio-temporal profile of the cell traction force in a wide field of view [8], and to determine where and how the force and stress are generated [9, 10].

In order to achieve the quantitative understandings of the resultant tissue morphogenesis, however, we need to elucidate the other mechanical factors as well, i.e., the mechanical properties that describe the relation between the deformation and forces. Although several pioneering works exist [11–14], our access to the mechanical properties is still limited. The characterization of these properties often requires exogenous manipulation of the tissue to induce deformation, but the procedure itself perturbs cell physiology and interferes with the tissue morphogenesis. Here, force measurement in a non-invasive manner gives a way to bypass this issue, and we can infer mechanical properties by associating spontaneous tissue deformation with the observed force dynamics.

In this study, we propose a reverse-engineering method to identify the mechanical properties, which is based on the combination of tissue mechanics modeling and statistical machine learning. Our strategy is to represent a cell monolayer as a continuum-mechanical system [15], and to use the passive and simultaneous observations of the deformation and traction force in order to compute the maximum likelihood estimate of the mechanical parameters. We formulated the inference as an inverse of the forward processes in which the mechanical properties and reaction force to the traction cause the tissue deformation. Our inference algorithm is based on the sequential updates of estimates; using the current model state and parameters, the mechanical model predicts the traction force field, and then the error feedback based on the observation is used to update the model state and parameters. Here, we applied our method to a cultured monolayer system to infer the elastic moduli from the collected tissue deformation and traction force data. To characterize the tissue deformation, we used velocity field of tissue motion, hereafter called tissue flow field.

## Materials and methods

### Cell culture and seeding

MDCK cells (strain II) were maintained in minimal essential medium (MEM; Invitrogen) supplemented with 10% fetal bovine serum (FBS; Equitech-Bio), GlutaMAX (Invitrogen), and 1 mM sodium pyruvate, in a 5% CO_2_ humidified incubator at 37 C°. According to a previously published protocol [9], 48 h before the image acquisition, 3 *μ*l drop of dense cell suspension (8 × 10^6^ cells/ml) was added to each dish containing the gel and 3 ml medium. Afterward, 3 h before the image acquisition, the medium was replaced by 3 ml CO_2_-independent medium supplemented with 10% FBS and GlutaMAX. For the myosin II inhibition, we added blebbistatin (Sigma Aldrich) at a final concentration of 25 *μ*M following the replacement of the medium.

### Traction force microscopy

Polyacrylamide gel substrates were prepared according to the previously published protocols [8, 9]. Briefly, the gel solution was prepared with 3% acrylamide, 0.25% bisacrylamide, 0.8% ammonium persulfate, 0.08% TEMED (Bio-Rad products), and 0.01% red fluorescent carboxylate-modified beads (0.5*μ*m diameter, Invitrogen). 20 *μ*l of this mixture was added to each dish and the samples were covered with glass cover slips with 18 mm diameter (Matsunami). After the polymerization, the surface was coated with type I collagen (Purecol, Advanced BioMatrix) using 4 *μ*M sulphosuccinimidyl-6-(4-azido-2-nitrophenylamino) hexanoate (Sulfo-SANPAH; Pierce). Young’s modulus of the gel was characterized by the conventional method using the Hertz equation [16], obtaining *E* = 2500±600 Pa. The Fourier-transform traction microscopy [8] was used to estimate traction force fields from bead displacement fields.

### Live imaging

Confocal imaging was conducted at 48 h after the seeding of the cells. We used FV10i-LIV (Olympus) to simultaneously acquire phase contrast images of the cells and fluorescent images of the beads. The trial period lasted for 6-10 h and the sampling rate was one frame per 5 min. After each trial, we removed the cells by the trypsinization and imaged the strain-free pattern of the fluorescent beads.

### Image analysis

To increase the field of view, we stitched tiled images by the Grid/Collection stitching plugin in Fiji [17]. Following this, the images at different time points were aligned to match the bead configurations in a cell-free region [18]. To obtain velocity fields in the phase contrast image and bead displacement fields, we adopted an advanced optical flow technique, which tracks changes between two images by matching the patterns of intensity and its gradient [19]. S1 Movie shows a representative result of the image analysis. For the tissue flow, we used images from subsequent time points, while for the bead displacement, we compared the stress-free image with each fluorescent image. The image resolution was 0.61*μ*m/pixel and the grid spacing of the vector fields was 14.7 *μ*m. Finally, the flow and force fields were down-sampled in space and time into Δ*x* = 29.4*μ*m and Δ*t* = 10 min.

### Continuum mechanical modeling

We adopted a continuum modeling of the monolayer mechanics, in which the deformation of the tissue, or strain, determines the stress [20, 21]. The cell monolayer was represented as a two-dimensional sheet, and therefore, we represented the stress as a symmetric matrix

$$\begin{array}{ccc} \sigma (x,y,t) & \equiv & (\begin{array}{cc} \sigma_{xx} & \sigma_{xy}\\ \sigma_{xy} & \sigma_{yy} \end{array})\\ & = & \pi I+\tilde{\sigma }. \end{array}$$ (1)

In the second line, we applied the deviatoric decomposition where the stress tensor is given as the summation of isotropic (the first term) and distortional (the second term) components. The strain tensor was also represented by a two-by-two matrix as

$$\begin{array}{c} \epsilon_{ij}\equiv \frac{1}{2}(\partial_{j}u_{i}+\partial_{i}u_{j}),\left(i,j=x,y\right), \end{array}$$ (2)

where (*u*_*x*_, *u*_*y*_) represents the displacement vector of the tissue from the stress-free state. In a linearly elastic material, the relationship between the stress and strain tensors becomes simply linear, meaning that the stress accumulates in response to the strain. However, the stress-free state of the living tissue can vary in time due to cell growth and death. Therefore, we adopted an alternative formulation using the strain rate tensor

$$\begin{array}{ccc} e(x,y,t) & \equiv & \dot{\epsilon }\\ \to e_{ij} & = & \frac{1}{2}(\partial_{j}v_{i}+\partial_{i}v_{j})\\ \to e & = & \frac{1}{2}\left(\nabla \cdot v\right)I+\tilde{e}, \end{array}$$ (3)

where *v* = (*v*_*x*_, *v*_*y*_) is the flow velocity vector in the tissue. In the last line, we applied the deviatoric decomposition. Although previous works suggested that anisotropic cell division can contribute the tissue mechanics [22, 23], we modeled the cell growth simply as isotropic and homogeneous expansion with the rate *D*_*g*_, which is partially supported by a previous report that cell division in the monolayer shows no particular orientation [24]. Since the observed total expansion of the tissue is the summation of the growth and the deformation-originated expansion, i.e., *D*_total_ = *D*_*g*_ + *D*_material_, the subtraction *D*_total_ − *D_g_* should appear in the stress-strain relation [15].

Taken together, our elastic model was written as

$$\begin{array}{c} \begin{array}{cc} \dot{\pi }\left(x,y,t\right) & =K(\nabla \cdot v\left(x,y,t\right)-D_{g})+\xi \left(x,y,t\right)\\ {\dot{\tilde{\sigma }}}_{xx}\left(x,y,t\right) & =2G{\tilde{e}}_{xx}\left(x,y,t\right)+\xi_{xx}\left(x,y,t\right)\\ {\dot{\tilde{\sigma }}}_{xy}\left(x,y,t\right) & =2G{\tilde{e}}_{xy}\left(x,y,t\right)+\xi_{xy}\left(x,y,t\right), \end{array} \end{array}$$ (4)

where *K* and *G* are the in-plane bulk and shear elastic moduli, respectively (in S1 Text, we derived the relation of the in-plane moduli to the conventional three dimensional moduli). *ξ*s are the stochastic terms representing random variables with Gaussian distribution that is not space or time-dependent. *D*_*g*_ is associated with the cell division interval *t*_div_ as *D_g_* = ln 2/*t*_div_, and we adopted *t*_div_ = 1 division per day. We found that essentially the same results are obtained by increasing or decreasing the rate of growth rate two times.

On the other hand, the tissue stress tensor and traction force vector were related through the force balance equation [25]

$$\begin{array}{c} \begin{array}{cc} -T_{x}\left(x,y,t\right) & =\frac{\partial \pi }{\partial x}+\frac{\partial {\tilde{\sigma }}_{xx}}{\partial x}+\frac{\partial {\tilde{\sigma }}_{xy}}{\partial y}+\eta_{x}\\ -T_{y}\left(x,y,t\right) & =\frac{\partial \pi }{\partial y}-\frac{\partial {\tilde{\sigma }}_{xx}}{\partial y}+\frac{\partial {\tilde{\sigma }}_{xy}}{\partial x}+\eta_{y}, \end{array} \end{array}$$ (5)

where *η*s are noises in the force quantification assumed to be normally-distributed, and we call them the observation noises.

### Inference algorithm

Here, we briefly describe the inference algorithm (the details of derivation is given in S1 Text). Let *Y* and Λ represent the collected spatio-temporal fields of traction force and tissue flow, respectively, and *X* represent the stress tensor field that was discretized in space and time according to *Y* and Λ. Additionally, let *θ* represent the model parameters. Then, our aim is to find such $\hat{\theta }$ that maximizes the log-likelihood:

$$\begin{array}{ccc} \ln L & \equiv & \ln p(Y|\Lambda ,\theta )\\ & = & \ln\int p(Y|X,\theta )p(X|\Lambda ,\theta )dX. \end{array}$$ (6)

Note that *p*(*X*|Λ, *θ*) and *p*(*Y*|*X*, *θ*) are corresponding to the stress evolution and force balance equations, i.e., Eqs 4 and 5, respectively. Since the integration w.r.t. *X* is analytically intractable, we adopted the expectation-maximization (EM) algorithm, which maximizes the lower-bound of the log-likelihood by executing the following E and M steps alternately [26].

(E-step) Estimate the stress fields by computing *p*(*X*|*Y*, Λ, θ*) with the Rauch-Tung-Striebel smoother [27], where θ* is a tentative estimate of the parameters.

(M-step) Compute the expected complete-data log-likelihood:

$$\begin{array}{c} Q\left(\theta \right)=E_{p(X|Y,\Lambda ,\theta^{*})}\left[\ln p\left(X,Y|\Lambda ,\theta \right)\right], \end{array}$$ (7)

and update the parameters through maximizing *Q*(*θ*).

Repeating the E and M steps, which offers monotonic increase and convergence of the likelihood. After the convergence, we obtain the maximum likelihood estimate of the parameters $\hat{\theta }$.

## Results

### Colony expansion assay

In order to collect the data on tissue deformation and force, we adopted a model system, Madin-Darby canine kidney (MDCK) epithelial cell monolayer, and analyzed it using the colony expansion assay [9]. We performed phase contrast imaging to measure the flow of cells in the monolayer and, simultaneously, traction force microscopy, in order to visualize the generated force using fluorescent beads embedded into the soft substrate.

Our inference algorithm used a mechanical model of the cell monolayer, i.e., a spatio-temporal model of mechanical stress within the tissue. Our mechanical model of the cell monolayer, represented by Eq 4 (see Materials and methods section), included two biophysical factors that are essential for the colony expansion: tissue elasticity and cell growth.

Elasticity is a basic property of a material, which resists the influence of an external force and shows a recoverable deformation. According to the previous studies [9, 14], we assumed the linear elasticity where the deformation is proportional to the force. Additionally, cellular growth supplies new cells into the tissue, and thereby promotes tissue expansion [28]. Our mechanical model also included stochasticity, which represents other mechanical processes such as viscosity, plasticity, active contractile force, and others.

**Fig 1:**
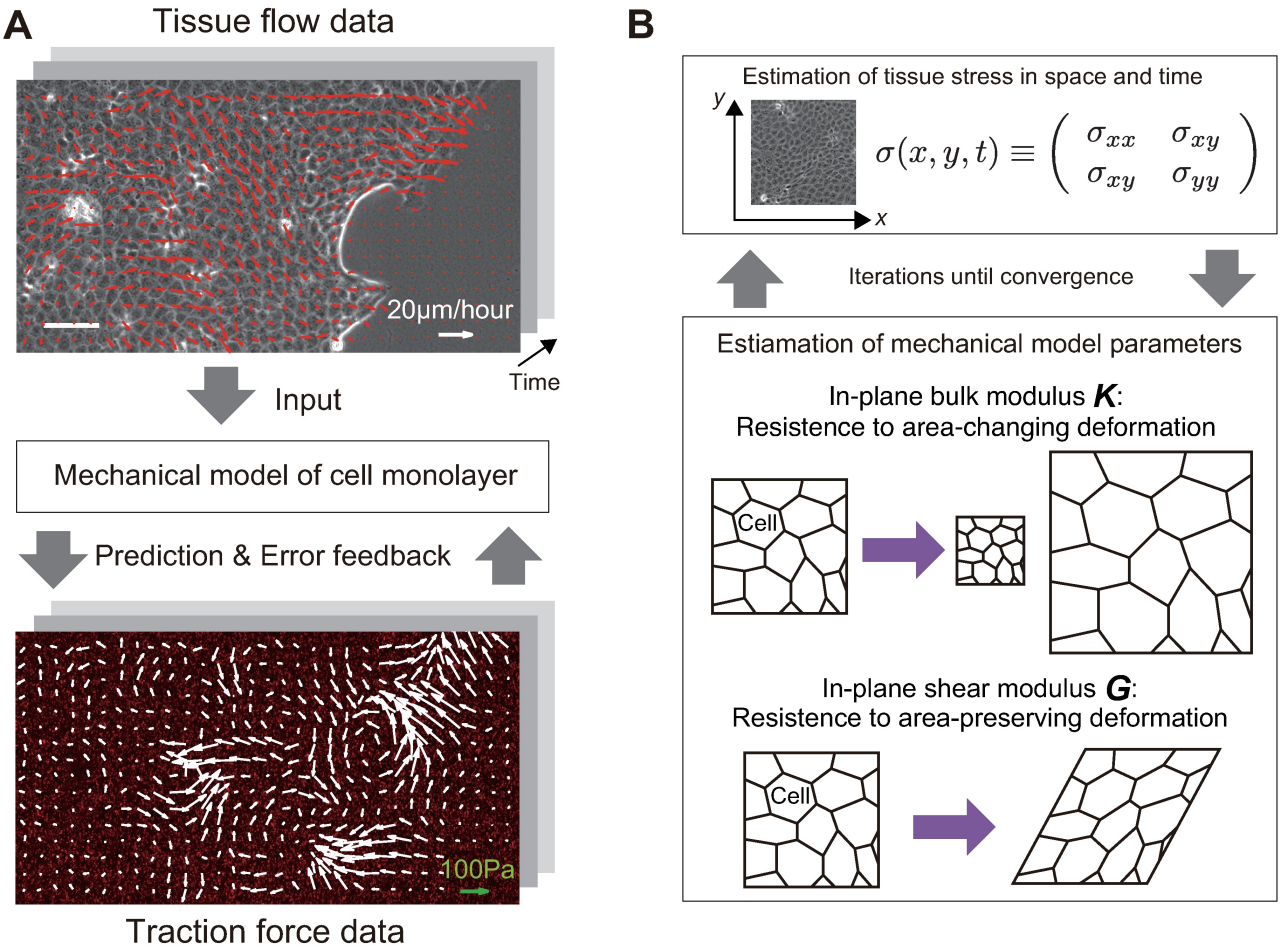
Overview of the inference algorithm. (A) A relation between our model and the obtained data. In a migrating cell monolayer, cells exert traction forces on the underlying substrate, in order to propel themselves forward. Since the cells adhere to the neighboring cells, the traction force introduces mechanical stress to the tissue and induces tissue flow. We simultaneously observed the velocity field in the tissue by the phase contrast imaging (top), and the traction force by measuring the displacement of fluorescent beads embedded into the soft substrate (bottom). The continuum-mechanical model, Eqs 4 and 5, quantitatively relates the tissue flow to the traction force by considering stress as the intermediate variable. At each time point, the model describes the stress evolution under the flow, and thus predicts the traction force at the next time step. Following this, the model receives the error feedback based on the difference between predicted and observed traction forces, so that the model state (the stress) is calibrated. (B) Schematic representation of the inference algorithm. Based on the procedure outlined in (A), the algorithm estimates the tissue stress tensor in space and time. Afterward, the model parameters are updated to reproduce the estimated stress dynamics. The procedure is repeated until the convergence.

This model, represented by Eq 4, had two mechanical parameters describing the elastic properties of the monolayer, the in-plane bulk modulus *K* and shear modulus *G*. The values of the bulk and shear moduli represent the resistance against area-changing and area-preserving deformation in the monolayer, respectively. Additionally, the elastic model contains the variance parameters for strength of the stochastic effects in the stress dynamics. Our inference algorithm, using the movie data showing tissue flow and traction force, estimated the values of these parameters (Fig 1A and 1B and “Materials and methods” section).

**Fig 2:**
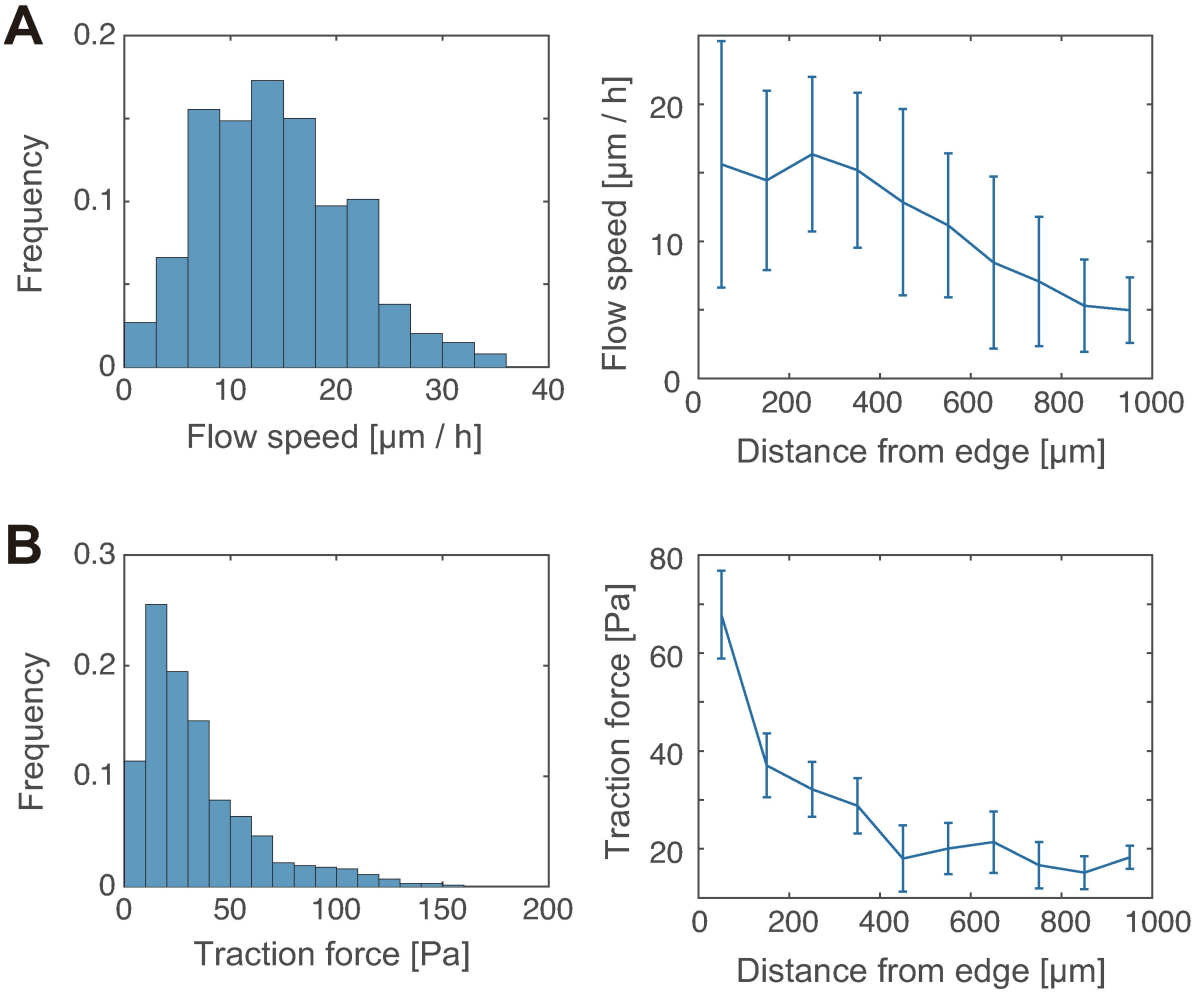
Quantification of tissue flow speed and traction force strength. (A) Histogram of the flow speed in the tissue within 500 *μ*m from the tissue edge (left; n >100 from three independent experiments), and the flow speed plotted against the distance from the tissue edge (right; n >10 at each data point). (B) The traction force data are presented in the same way as the flow speed data in (A).

We found that the flow speed of the tissue at the periphery was approximately 10-30 *μ*m/h (Fig 2A), the strength of the traction force was distributed around 10-100 Pa (Fig 2B), and both flow speed and force strength decreased monotonically along the distance from the edge (Fig 2A and 2B). These results are consistent with the previous observations [4, 8, 29].

### Traction force field forecast

**Fig 3:**
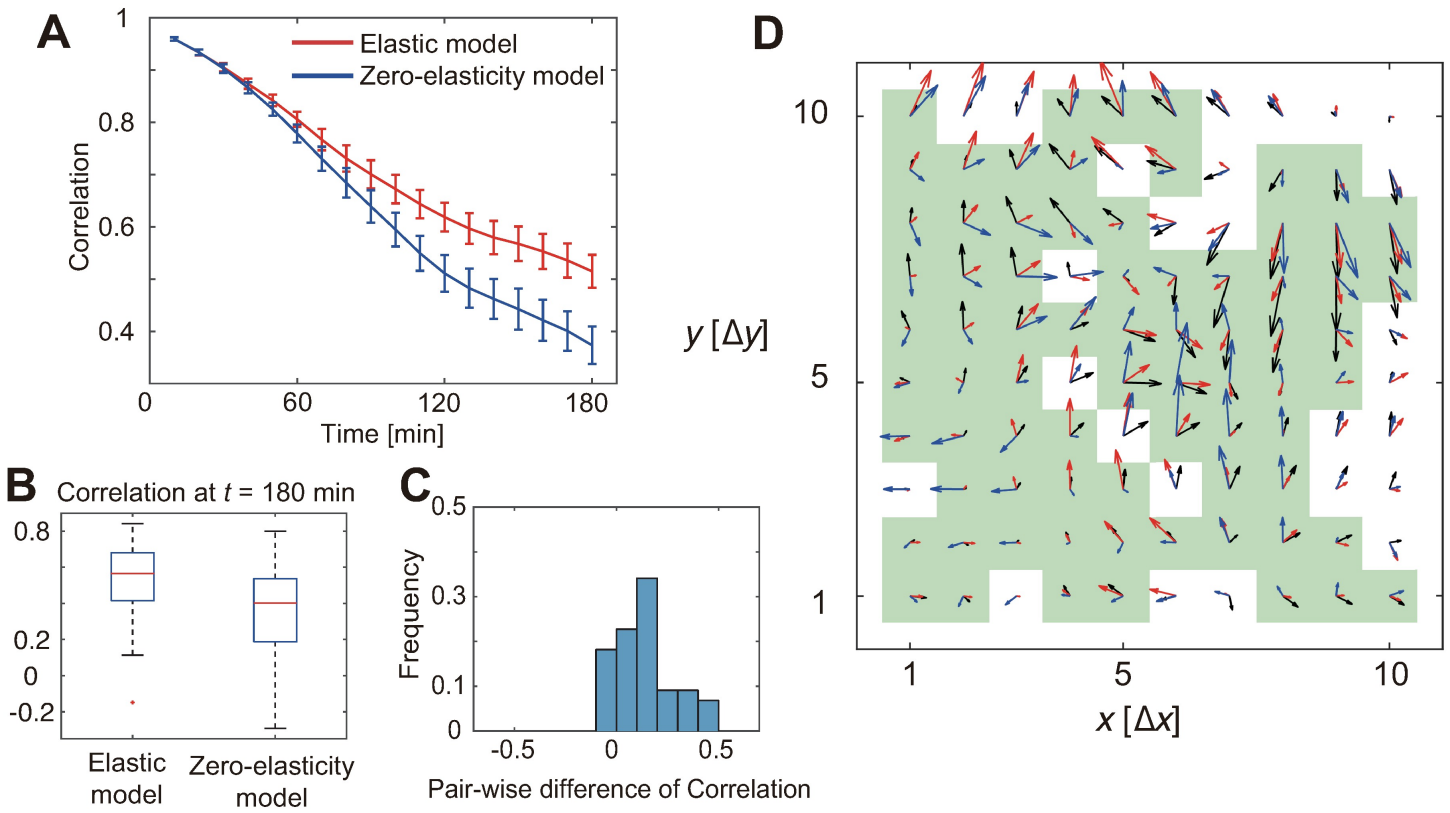
Traction force forecast. (A) Correlation between the observed and predicted traction force fields. The mean and standard error (S.E.; n = 44 from three independent experiments) are plotted against the time lapse. Red and blue lines show the forecasts by the elastic model and the zero-elasticity model, respectively. (B) The predictive performance at the last time point. Each blue box shows upper and lower quartiles. The horizontal red, upper black, and lower black lines indicate the median, maximum, and minimum values, respectively, disregarding the outliers. (C) The difference in the data reproducibility, in terms of the correlation of the actual data and the reproduced data, between the elastic and zero-elasticity models. The histogram was taken in a sample-wise manner; that is, each point corresponds to a single movie. The elastic model shows statistically significant superiority (p <10^−4^, Wilcoxon signed-rank test). (D) A representative image of force fields at the last time point (t = 180 min). The black arrows show observed force vectors, while the red and blue arrows show forecasts by the elastic and zero-elasticity models, respectively. The green squares indicate grid points where the forecast by the elastic model was better than that by the zero-elasticity model. Δ*x* = Δ*y* = 29.4 *μ*m.

We computed maximum likelihood estimates of the parameters in our elastic model from the collected data. For this estimation, the model state, i.e., the tissue stress field, was corrected by the current traction force data at each time point in the movie sequence; following this, the model was numerically simulated, using the tissue flow data, in order to predict the traction force field at the following time point. As a result, even though the model inference was based on the one-step prediction (Δ*t* = 10 min), we found that the estimated model can provide a long-term forecast (>1 h) without corrected by the traction force data. To quantitatively demonstrate this result, we divided each movie data on tissue flow and traction force, into two parts in time: The earlier, training data and the following test data. Using the training data, the inference algorithm was used to estimate the model parameters and the stress field in the monolayer, and then this model was examined in terms of the forecast accuracy for future force fields by using the test data. As a quantitative measure, we employed the correlation between the forecasted and observed force vector fields:

$$\begin{array}{c} R=\frac{\left\langle T_{\text{forecast}}\cdot T_{\text{data}}\right\rangle }{\left\langle |T{|}_{\text{forecast}}\cdot |T{|}_{\text{data}}\right\rangle }, \end{array}$$ (8)

where 〈⋅〉 represents an average over all spatial grid points. The correlation plotted against time is represented in Fig 3A. As shown, the forecast provided by the elastic model was highly correlated even 3 h after the initiation of the test part. For comparison, we adopted a null-hypothetical, zero-elasticity model, where *K* = *G* = 0 (Fig 3A). In Fig 3B, the correlation in both models at the last time-point in the test part, the long-term forecast accuracy, is shown. These results demonstrate that our data-driven elastic model showed better forecast which is clearer especially in longer time forecast. We also computed the difference of correlation between the models in a sample-wise manner (Fig 3C), and confirmed statistically-significant superiority of the elastic model compared with the zero-elasticity model (*p* < 10^−4^, Wilcoxon signed-rank test). Representative forecasted and observed traction forces at the last time point are shown in Fig 3D. Therefore, despite of its simplicity, our data-driven elastic model captured the stress evolution in the tissue expansion by the estimated bulk (*K*) and shear (*G*) moduli.

### Estimation of monolayer elastic moduli

**Fig 4:**
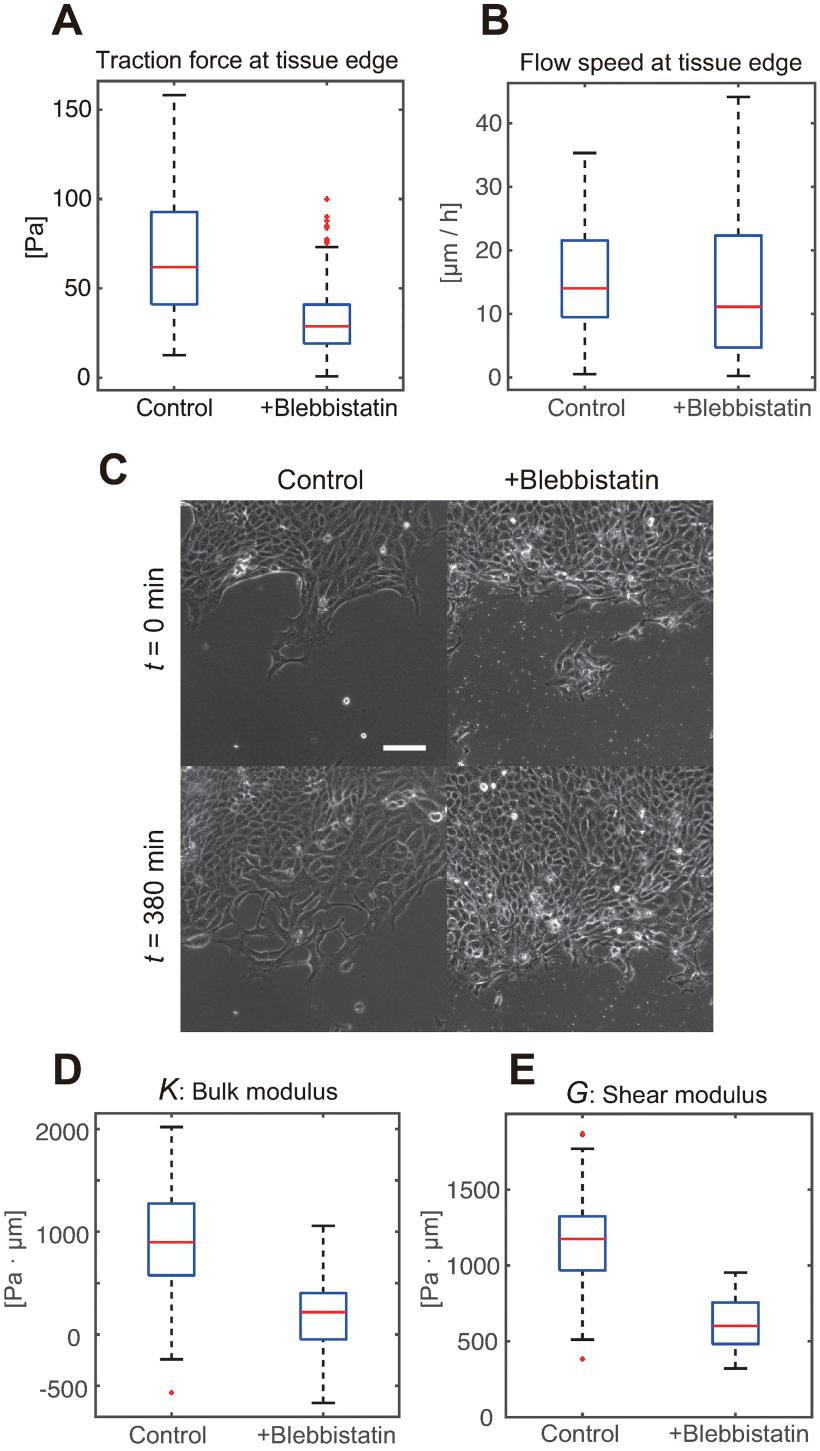
Estimated elastic moduli of the MDCK cell monolayer with/without myosin inhibition. (A) Traction force strength at the tissue edge. The traction force vectors within 100 *μ*m from the tissue edge were collected (n >100 in both control and blebbistatin treatment groups). Each blue box shows upper and lower quartiles. The horizontal red, upper black, and lower black lines indicate the median, maximum, and minimum values, respectively, expect for the outliers. (B) Flow speed at the tissue edge computed as the traction force strength. The sheet migration under the motor inhibition did not slow down despite of the weakened traction force. (C) Phase contrast images of the advancing front, exemplifying that the advancement of the blebbistatin-treated tissue did not slow down (scale-bar = 100 *μ*m). (D) Boxplots show the bulk modulus estimated by our method, based on the movies obtained in the standard setting (control, n = 44 from three independent experiments), and from tissues treated with blebbistatin (+blebbistatin, n = 9 from two independent experiments). (E) The shear modulus results are presented in the same way as the bulk modulus results in (D).

Next, we examined if the elastic moduli are different in the tissues treated with blebbistatin, a myosin inhibitor. Previous studies showed that the inhibition of the molecular motors considerably reduces the traction force strength, which is expected. However, this inhibition does not slow down the tissue expansion rate [6], indicating the alterations in tissue mechanical properties. By comparing the elastic moduli estimated from a different dataset from blebbistatin-treated tissues with those estimated under the standard conditions (Fig 4A, 4B and 4C), we obtained the results consistent with those obtained in previous experiments. We observed a significant decrease in the elastic moduli associated with the treatment (Fig 4D and 4E). In the standard experimental setting, both moduli were within the order of the magnitude of ∼ 10^3^Pa · *μ*m, while myosin inhibition induced several-fold reduction in the elastic modulus values, i.e., softening (*p* < 0.01, U-test). Note that the estimated moduli are not guaranteed to have positive values because unmodeled monolayer mechanics, such as viscosity and anisotropic tissue growth, might affect the stress dynamics. In fact, the estimated moduli from myosin-inhibited tissues have frequently shown negative values, suggesting that the elasticity effect was no longer dominant over unmodeled effects due to the softening.

**Fig 5:**
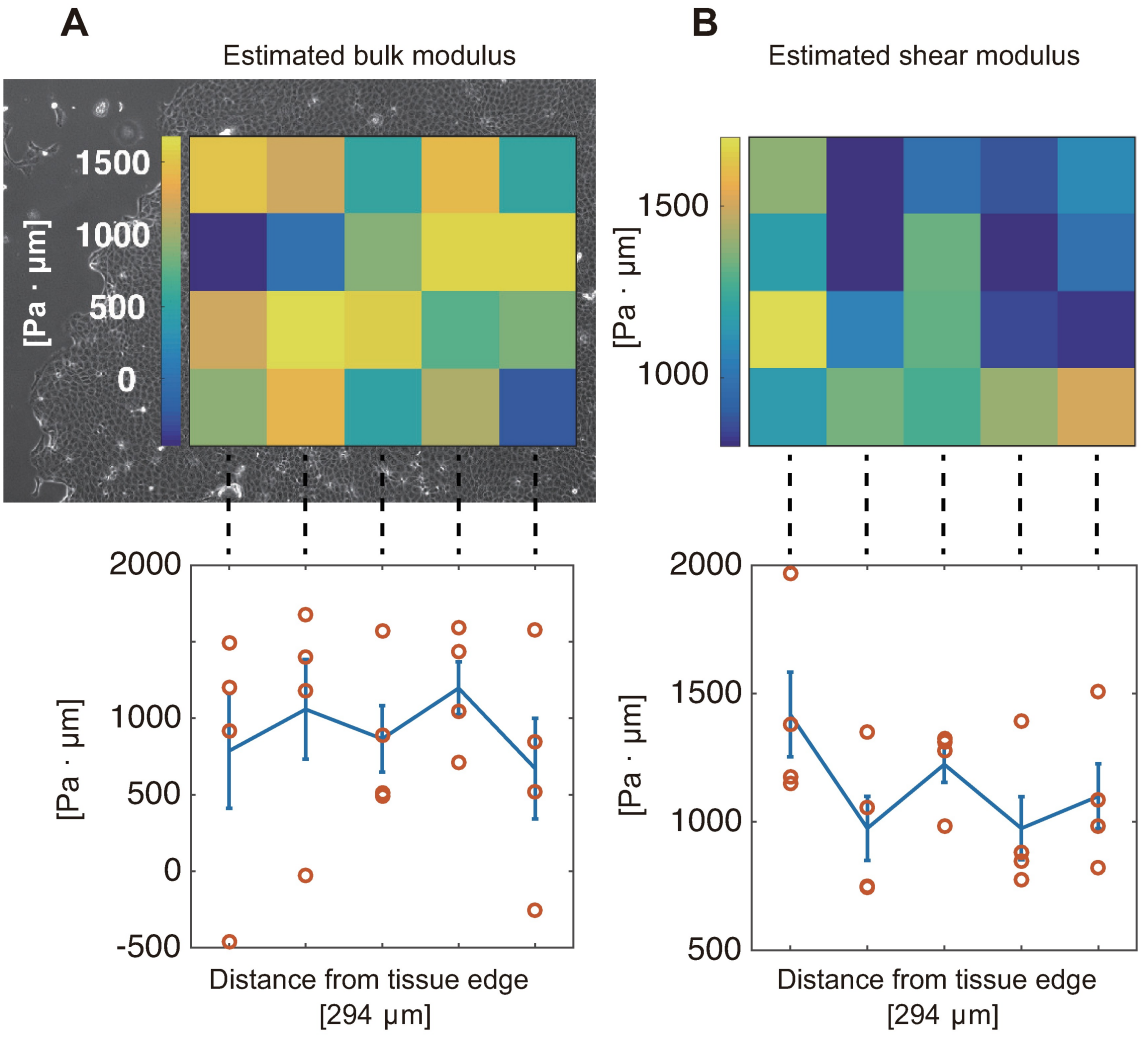
Spatial distribution of the estimated elastic moduli. Notice that each estimate comes from the movie patch with 294*μ*m-by-294*μ*m size. (A) The heatmap of estimated values of bulk modulus is overlaid on the corresponding phase contrast image of cell monolayer (top), and the mean and S.E. are plotted against the distance from the edge (bottom), in which no dependence on the distance has been observed. (B) The shear modulus results are presented in the same way as the bulk modulus results in (A). No dependence on the distance from the edge either.

Finally, we assessed the spatial distribution of the elastic moduli. When considering difference in the flow speed and force strength dependent on the distance from the tissue edge (Fig 2), we expected the mechanical properties that would correlate with this distance. However, as shown in Fig 5, we found that the estimated moduli are homogenous along with the distance.

## Discussion

In this study, we developed a non-invasive reverse-engineering method for the elucidation of the mechanical properties of cell monolayers, based on the combination of the simulation of tissue mechanics and statistical machine learning. The method allowed us to estimate the in-plane bulk and shear moduli of a cell monolayer in motion. In the inverse mechanics algorithm, the mechanical model predicted the traction force based on the tissue flow data, and received the error feedback in order to update the model state and parameters. Upon the application of this method to the tissue expansion of MDCK cell monolayer, we found that the data-driven elastic model outperforms the zero-elasticity model, the null-hypothesis model, in the long-term forecast of the traction force fields. Although the viscoelastic properties of living tissues still represent a controversial subject, especially under the slow time-scale of spontaneous deformation, our results indicate that the elasticity contributes to the stress evolution during tissue expansion. Note that the elasticity of the tissues does not necessarily originate from cellular materials, but effectively from the mechano-responsive cellular behaviors, such as altered cytoskeletal activity [30, 31]. This suggests that the mechanical properties of tissues can largely depend on cell physiology, and therefore, it would be interesting to apply our method to different tissue types and environments.

The elastic properties of the MDCK cell monolayer were investigated in several previous studies [14, 31]. Our estimated moduli were shown to be two orders of magnitude smaller than the results of the previous study, which employed a freely-suspended monolayer, but our moduli were consistent with the previous results on an advancing monolayer attached to a soft substrate; the later setting was similar to ours. The latter results were obtained by comparing the spatially-integrated strain and traction force in a uniform one-dimensional tissue expansion. Therefore, our method can be interpreted as a generalization of the previous approach, an expansion from scalar-value-based to two-dimensional-field-based strategy, and the consistency between the obtained results increases the reliability of these approaches. Based on the obtained results, we can speculate that an advancing monolayer becomes much softer than a static monolayer, which may promote the expansion during wound healing and embryogenesis. However, this should be further validated.

The estimated values for bulk and shear moduli are similar to each other (∼ 1 kPa⋅*μ*m). If we assume a simple three-dimensional elasticity model (See Eqs. S25 and S26 in S1 Text), the results indicate that the in-plane Poisson’s ratio *ν*_in-plane_ is close to zero. It is consistent with a previous work [14] where the authors measured the strain in cell monolayer under exogenous forces. Their result indicates *ν*_in-plane_ ~ 0 while *ν*_out-of-plane_ > 0. On the other hand, reported values for Poisson’s ratio of living tissues are mainly around 0.5 [32]. Currently, we are not sure where the discrepancy came from. A possibility is that our model was too simple to determine Poisson’s ratio reliably. Although the linear elasticity model has been successfully applied to the MDCK cell monolayer [14, 31], it is still far from forecasting the force/stress dynamics accurately, as shown in Fig 3A. We hope that more elaborate modeling of elasticity as in [33, 34] would be useful to resolve the issue.

We observed a several-fold reduction of both elastic moduli in the blebbistatin-treated tissues. The softening of the tissue may compensate for the several-fold reduction in the traction force strength at the advancing edge, so that the expansion speed may remain the same despite the weakened force. Note that cell/tissue softening upon myosin inhibition has been observed in previous investigations, using different cell types, which indicates that this is a universal phenomenon [31, 35, 36]. However, the molecular-level mechanism remains elusive and calls for theoretical advances in cytoskeletal biophysics [37]. Furthermore, such better biophysical understandings would be beneficial to improve the accuracy of estimated moduli. We found that the estimated moduli from myosin-inhibited tissues (Fig 4D and 4E) have occasionally taken negative values. This suggests that unmodeled mechanical dynamics, which are currently represented by zero-mean noise terms in Eq 4, were not negligible compared with the elastic stress evolution. Therefore, the current estimation results from the softened tissues seem not quantitatively accurate, and better modeling is required to get reliable values.

We assessed the spatial distribution of the elastic moduli, which has not been investigated so far for the advancing monolayers. We found no particular spatial character in the moduli. Although this seems to support the homogeneity assumption underlying recent analyses [9, 25], the estimated moduli exhibited significant variation between the data. Although this variation may originate from the actual differences in the elastic properties, another possibility is that the modeling of the tissue mechanics is performed with insufficient details. In particular, we ignored the active stress generation on the apical side because we focused exclusively on the traction force on the basal side. Therefore, if the actomyosin cytoskeleton on the apical side affects tissue deformation as observed in *Drosophila* pupal development [2, 38], our estimation may have been significantly perturbed. Because of this, further investigations of these variations will be obtained by observing the actomyosin activity and incorporating the data into the model.

Our mechanical model does not explicitly include any viscous effect for simplicity. However, it is still informative to extend the model to include such an effect and perform model comparison. To this end, we adopted the Maxwell model that can account for stress relaxation through cell rearrangement [11, 15]. We found that the extended model exhibited overfitting on our data, while the estimated moduli took similar values to those from the elastic model (See Supporting Results section in S1 Text). The latter result suggests robustness of the estimates of elastic moduli.

### Conclusion

The framework we presented in this study would benefit from the advances in force measurement. For example, although the traction force microscopy is applicable only to *in vitro* tissues, *in vivo* measurement techniques are being actively developed [38, 39]. We can apply our reverse-engineering method to the *in vivo* measuring by modifying the model of the observation process, Eq 5 in our case. Additionally, another interesting direction would be to use a more elaborate model of tissue mechanics, in particular, by directly including cellular processes such as cell division [40, 41]. We hope that, with the advancements in the technology of force/stress measurement, our method may assist further understanding of the mechanics underling tissue development and maintenance.

## Supporting information

S1 Text Supporting methods, results, and discussion. (PDF)

S1 Movie Dynamics of tissue flow and traction force. A representative movie of the velocity fields in the tissue (left) and the traction force fields (right) is presented. Scale bar indicates 100*μ*m.
(MOV)
