# Supplementary material for: Inverse tissue mechanics of cell monolayer expansion
Source: PLoS Comput Biol. 2018 Mar 1;14(3):e1006029. doi: 10.1371/journal.pcbi.1006029 (PMC5849322; doi:10.1371/journal.pcbi.1006029)
Supplement: S1 Text — (PDF) [file pcbi.1006029.s001.pdf]

S1 Text: Inverse tissue mechanics of cell monolayer expansion

Y. Kondo, K. Aoki, and S. Ishii

CONTENTS

Supporting Methods 1

Supporting Results 3

Supporting Discussion 4

References 5

SUPPORTING METHODS

State-space model representation

In order to increase the convenience of deriving the inference algorithm, we converted the mechanical model and the force balance equation, Eqs. 4 and 5 in the main text, respectively, into a form of state-space model:

$$\boldsymbol{x}^{t+1} = A^t \boldsymbol{x}^t + \boldsymbol{b}^t + \boldsymbol{\epsilon}^t, \tag{S1}$$

$$\boldsymbol{y}^t = C\boldsymbol{x}^t + \boldsymbol{\eta}^t, \tag{S2}$$

where $\boldsymbol{x}^t$ is a vector that represents the model state at time point t, A^t is a time-dependent parameter matrix, $\boldsymbol{b}^t$ is an input vector, $\boldsymbol{\epsilon}^t$ is a vector of independent Gaussian noises with variance V_ϵ, $\boldsymbol{y}^t$ is an observation vector, C is a matrix that represents the observation process, and $\boldsymbol{\eta}_t$ is a vector of independent Gaussian noises with variance V_η. Eqs. S1 and S2 are called state equation and observation equation, respectively.

In particular, we discretized the two-dimensional stress field in space and time, and then converted the discretized field into a column vector as

$$\boldsymbol{x}^t = (\boldsymbol{s}^t_{11}, \boldsymbol{s}^t_{12}, \boldsymbol{s}^t_{13}, ..., \boldsymbol{s}^t_{21}, \boldsymbol{s}^t_{22}, ..., \boldsymbol{s}^t_{NN})^\top, \tag{S3}$$

where $\boldsymbol{s}^t_{nm}$ represents $(\pi^t, \tilde{\sigma}^t_{xx}, \tilde{\sigma}^t_{xy})$ at $(n\Delta x, m\Delta y, t)$. In Eq. 4, we employed the commonly-used upper-convected time derivative as

$$\dot{\pi} \equiv \frac{\partial \pi}{\partial t} + v \cdot \nabla \pi - \frac{1}{2}\mathrm{Tr}(e\sigma + \sigma e), \tag{S4}$$

$$\begin{aligned}\dot{\tilde{\sigma}} \equiv\ & \frac{\partial \tilde{\sigma}}{\partial t} + v \cdot \nabla \tilde{\sigma} + (\tilde{\sigma}\omega - \omega\tilde{\sigma}) \\ & -[(e\sigma + \sigma e) - \frac{1}{2}\mathrm{Tr}(e\sigma + \sigma e)I],\end{aligned} \tag{S5}$$

where ω is the vorticity tensor. For the first terms in Eqs. S4 and S5, we adopted the forward difference for the time derivatives. For the following terms, we adopted the central difference for space derivatives, so that the terms were represented as $A^t_1 \boldsymbol{x}^t$. At the boundaries, forward or backward difference was adopted instead of central difference. The other terms could be expressed as $A^t_2 \boldsymbol{x}^t$ with a block diagonal matrix A^t_2 in which each diagonal block for $\boldsymbol{s}^t_{nm}$ was

$$\begin{pmatrix} \nabla\cdot\boldsymbol{v} & 2\tilde{e}_{xx} & 2\tilde{e}_{xy} \\ 2\tilde{e}_{xx} & \nabla\cdot\boldsymbol{v} & 0 \\ 2\tilde{e}_{xy} & 0 & \nabla\cdot\boldsymbol{v} \end{pmatrix} - \begin{pmatrix} 0 & 0 & 0 \\ 0 & 0 & -2\omega_{xy} \\ 0 & 2\omega_{xy} & 0 \end{pmatrix}. \tag{S6}$$

The elastic terms in Eq. 4 were simply expressed as

$$\begin{aligned}\boldsymbol{b}^t_1 &\equiv \boldsymbol{\alpha}^t \circ \boldsymbol{\lambda}^t, \\ &\equiv (K, 2G, 2G, ...)^\top \circ (\nabla\cdot\boldsymbol{v} - D_g, \tilde{e}_{xx}, \tilde{e}_{xy}, ...)^\top,\end{aligned} \tag{S7}$$

where '$\circ$' is the Hadamard product (i.e., element-wise multiplication). Taken together, by setting $A^t = I - \Delta t(A_1^t + A_2^t)$ and $\boldsymbol{b}^t = \Delta t \boldsymbol{b}_1^t$, and adding the Gaussian noise, we obtained the state equation in the form of Eq. S1. Concerning the force balance equation, Eq. 5, we converted the traction force vector field $T(x, y, t)$ into a column vector as follows:

$$\boldsymbol{y}^t = (\boldsymbol{f}_{11}^t, \boldsymbol{f}_{12}^t, \boldsymbol{f}_{13}^t, ..., \boldsymbol{f}_{21}^t, \boldsymbol{f}_{22}^t, ..., \boldsymbol{f}_{NN}^t)^\top, \tag{S8}$$

where f_{nm}^t represents $(-T_x, -T_y)$ at $(n\Delta x, m\Delta y, t)$. Following this, the discretization of the space derivatives of the stress with the central difference scheme led to $C\boldsymbol{x}^t$.

Prior distribution

Concerning the initial condition for $\boldsymbol{x}^t$, we assumed the Gaussian distribution $\boldsymbol{x}^0 \sim \mathcal{N}(\boldsymbol{\mu}, V_\mu I)$, where I is the identity matrix. However, we found that estimating $\boldsymbol{\mu}$ freely caused extremely slow convergence, likely because the partial observation in the force balance equation was not sufficient to fully determine the initial stress field. Therefore, motivated by a previous study on estimating stress in the monolayer [S1], we imposed the prior over $\boldsymbol{\mu}$ as

$$p(\boldsymbol{\mu}) \propto e^{-\frac{\|\boldsymbol{\mu}\|_2^2}{2V_\mu}}, \tag{S9}$$

which effectively accelerated the convergence.

Derivation of inference algorithm

Let X denote $\{\boldsymbol{x}^0, ..., \boldsymbol{x}^{T-1}\}$, and Y and Λ were used in the same manner. Let θ denote all of the parameters, i.e., $\{K, G, V_\epsilon, V_\eta, \boldsymbol{\mu}, V_0\}$. Given Y and Λ, representing the traction force and tissue flow movie data, respectively, the log likelihood was represented as

$$\begin{aligned} \ln L &\equiv \ln p(Y|\Lambda, \theta) \\ &= \ln \int p(Y|X, \theta) p(X|\Lambda, \theta) dX. \end{aligned} \tag{S10}$$

The aim was to find such $\hat{\theta}$ that maximizes the log likelihood. Since the integration w.r.t. X is analytically intractable, we adopted the expectation-maximization (EM) algorithm, which maximizes the lower-bound of the log likelihood by executing the following E and M steps alternately.

In the E step, the Rauch-Tung-Striebel smoother [S2] computed $p(\boldsymbol{x}^t|Y, \theta^*)$ where θ^* is a tentative estimate of the model parameters. Using this procedure, we calculated $\mathbb{E}[\boldsymbol{x}^t]$, $\mathbb{E}[\boldsymbol{x}^t \boldsymbol{x}^{t\top}]$, and $\mathbb{E}[\boldsymbol{x}^{t+1} \boldsymbol{x}^{t\top}]$, required for the next M step.

In the M step, we updated θ by maximizing the expected complete-data log-likelihood function with the prior at the initial condition,

$$\begin{aligned} Q &\equiv \mathbb{E}_{p(X|Y,\Lambda,\theta^*)}[\ln p(X, Y|\Lambda, \theta)] + p(\boldsymbol{\mu}) \\ &= -N^2 T \ln V_\eta - \frac{1}{2V_\eta} \sum_{t=0}^{T-1} \mathbb{E}[\|\boldsymbol{y}^t - C\boldsymbol{x}^t\|_2^2] \\ &\quad - \frac{3N^2(T-1)}{2} \ln V_\epsilon - \frac{1}{2V_\epsilon} \sum_{t=0}^{T-2} \mathbb{E}[\|\boldsymbol{x}^{t+1} - A^t \boldsymbol{x}^t - \boldsymbol{b}^t\|_2^2] \\ &\quad - \frac{3N^2}{2} \ln V_0 - \frac{1}{2V_0} \mathbb{E}[\|\boldsymbol{x}^0 - \boldsymbol{\mu}\|_2^2] - \frac{\|\boldsymbol{\mu}\|_2^2}{2V_\mu}. \end{aligned} \tag{S11}$$

Note that, in an N-by-N grid space, the dimensions of $\boldsymbol{x}^t$ and $\boldsymbol{y}^t$ are $3N^2$ and $2N^2$, respectively. By taking the

gradient of Q w.r.t. θ and setting it to zero, we obtained the closed-form solutions for the parameter update as

$$K = \frac{\sum_{t=0}^{T-2} \mathrm{Tr}(I_1 \circ H^t)}{\Delta t \sum_{t=0}^{T-2} \mathrm{Tr}(I_1 \circ \boldsymbol{\lambda}^t \boldsymbol{\lambda}^{t\top})}, \tag{S12}$$

$$G = \frac{\sum_{t=0}^{T-2} \mathrm{Tr}(I_2 \circ H^t)}{2\Delta t \sum_{t=0}^{T-2} \mathrm{Tr}(I_2 \circ \boldsymbol{\lambda}^t \boldsymbol{\lambda}^{t\top})}, \tag{S13}$$

$$V_\epsilon = \frac{\sum_{t=0}^{T-2} \mathrm{Tr} E_1^t}{3N^2(T-1)}, \tag{S14}$$

$$V_\eta = \frac{\sum_{t=0}^{T-1} \mathrm{Tr} E_2^t}{2N^2 T}, \tag{S15}$$

$$\mu = \frac{\mathbb{E}[\boldsymbol{x}^0]}{1 + V_0/V_\mu}, \tag{S16}$$

$$V_0 = \frac{\mathrm{Tr}(\mathbb{E}[\boldsymbol{x}^0 \boldsymbol{x}^{0\top}] - \mathbb{E}[\boldsymbol{x}^0]\boldsymbol{\mu}^\top - \boldsymbol{\mu}\mathbb{E}[\boldsymbol{x}^{0\top}] + \boldsymbol{\mu}\boldsymbol{\mu}]^\top)}{3N^2}, \tag{S17}$$

where $H^t = (\mathbb{E}[\boldsymbol{x}^{t+1}] - A^t \mathbb{E}[\boldsymbol{x}^t])\boldsymbol{\lambda}^{t\top}$, $I_1(I_2)$ represents the indices of isotropic (deviatoric) components as

$$I_1 = \mathrm{diag}(1, 0, 0, 1, 0, 0, ...), \tag{S18}$$

$$I_2 = \mathrm{diag}(0, 1, 1, 0, 1, 1, ...), \tag{S19}$$

and

$$E_1^t = \mathbb{E}\left[(\boldsymbol{x}^{t+1} - A^t \boldsymbol{x}^t - \boldsymbol{b}^t)(\boldsymbol{x}^{t+1} - A^t \boldsymbol{x}^t - \boldsymbol{b}^t)^\top\right], \tag{S20}$$

$$E_2^t = \mathbb{E}\left[(\boldsymbol{y}^t - C\boldsymbol{x}^t)(\boldsymbol{y}^t - C\boldsymbol{x}^t)^\top\right]. \tag{S21}$$

The updates of the variances V_ϵ, V_η, and V_0 require the updated values of K, G, and μ.

We set the initial values of the parameters as $K = 0$, $G = 0$, $V_\epsilon = 10^4 \Delta t$, $\mu = 0$. We fixed the other parameter values as $V_\eta = 10^2$, $V_0 = 10^4$, and $V_\mu = 10^5$ for numerical stability. The number of grid points of the tissue flow and traction force fields was 10-by-10. The time step size was $\Delta t = 10$ min.

Constraint on elastic moduli

We could impose prior distributions on the mechanical parameters, K and G, in order to prevent overfitting to noises and limit the parameter values within reasonable ranges. However, we have not used such a prior in this study, because it might mitigate pathological estimation results coming from insufficient mechanical modeling, which makes model validation unclear.

Nevertheless, imposing such a constraint is quite beneficial in practice, and thus let us discuss a few ways to implement the idea in our framework. Firstly, the easiest way for imposing positivity of the moduli is to use a uniform distribution $[0, \infty]$. The estimation can be achieved simply by applying the rectified linear function, $f(x) = \max(0, x)$, to the parameter update. Secondly, when we have expected values for the moduli, e.g., from a literature, an option is to use a factorized Gaussian prior, $p(K, G) = N(K|K_0, V_K) \times N(G|G_0, V_G)$. In this case, since $\frac{\partial}{\partial K} \log p(K, G)$ is a linear function w.r.t. K, we are still able to compute the closed-form solution to update K. The same is true for G. However, with more complex priors, it would be intractable to analytically compute the parameter update equations. In that cases, the approximate Bayesian learning such as the variational Bayes would be useful.

SUPPORTING RESULTS

Estimation results for Maxwell model

A possible shortcoming of our elastic model is that the model does not include stress relaxation through, e.g., cell rearrangement [S3]. Thus, for model comparison, we adopted the Maxwell model that can account for the effect. We

can write the model equations for stress evolution simply by adding a few terms to Eqs. 4 as

$$\begin{aligned} \dot{\pi}(x,y,t) &= ... \\ \dot{\tilde{\sigma}}_{xx}(x,y,t) &= ... - \tau^{-1}\tilde{\sigma}_{xx} \\ \dot{\tilde{\sigma}}_{xy}(x,y,t) &= ... - \tau^{-1}\tilde{\sigma}_{xy}, \end{aligned} \tag{S22}$$

where τ^{-1} is the stress relaxation rate. Since the modified equations are linear w.r.t. the new parameter τ^{-1}, we are still able to obtain the analytical solutions for the parameter update in the EM algorithm. Fig. A shows the estimated parameter values for the Maxwell model. The values of estimated moduli were close to those in the elastic model, which indicates robustness of the estimation. On the other hand, the estimated values of stress relaxation rate were distributed around $\tau^{-1} \sim 0.1$ hour^{-1}, which is not unrealistic according to previous studies, but the variation among samples was too large to determine the typical time scale of stress relaxation. More importantly, as shown in Fig. B, the Maxwell model did not exhibit better performance in the traction force forecast compared with the elastic model, despite the increased complexity. Those results indicate that the Maxwell model exhibited overfitting on our data.

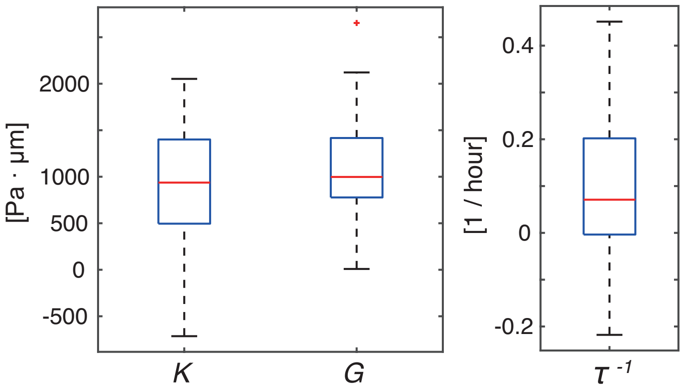

FIG. A. Estimated elastic moduli (left) and stress relaxation rate (right) for the Maxwell model from the same data as in Fig. 3.

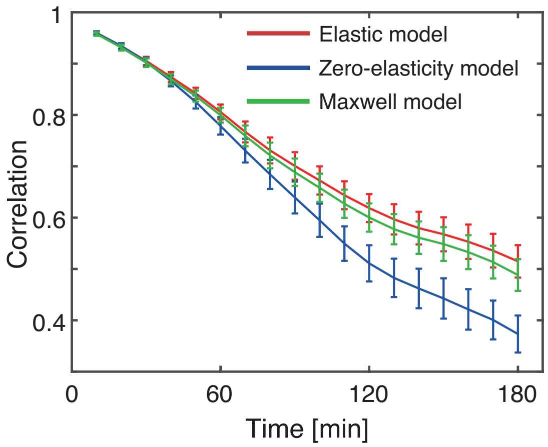

FIG. B. The forecast performance of Maxwell model is presented in the same way as the other models in Fig. 3A.

SUPPORTING DISCUSSION

Relation of in-plane and three-dimensional moduli

Here we associate our in-plane elastic moduli with the conventional three-dimensional moduli. In this section, we express the in-plane moduli with the subscripts as $K_{\text{in-plane}}$ and $G_{\text{in-plane}}$ in order to avoid confusion with their

three-dimensional counterparts. We assume that the cell sheet is a transversely isotropic elastic material, i.e., the mechanical properties are isotropic in the in-plane direction of cell sheet but can be different in the normal direction along with the apical-basal axis. Thus, using the common notation where E, G and ν are Young's modulus, the shear modulus, and Poisson's ratio, respectively, we describe the three-dimensional stress-strain relation as

$$\begin{pmatrix} \epsilon_{xx} \\ \epsilon_{yy} \\ \epsilon_{zz} \\ 2\epsilon_{yz} \\ 2\epsilon_{zx} \\ 2\epsilon_{xy} \end{pmatrix} = \begin{pmatrix} 1/E_1 & -\nu_1/E_1 & -\nu_2/E_2 & 0 & 0 & 0 \\ -\nu_1/E_1 & 1/E_1 & -\nu_2/E_2 & 0 & 0 & 0 \\ -\nu_2/E_2 & -\nu_2/E_2 & 1/E_2 & 0 & 0 & 0 \\ 0 & 0 & 0 & 1/G_2 & 0 & 0 \\ 0 & 0 & 0 & 0 & 1/G_2 & 0 \\ 0 & 0 & 0 & 0 & 0 & \frac{2(1+\nu_1)}{E_1} \end{pmatrix} \begin{pmatrix} \sigma_{xx} \\ \sigma_{yy} \\ \sigma_{zz} \\ \sigma_{yz} \\ \sigma_{zx} \\ \sigma_{xy} \end{pmatrix}, \tag{S23}$$

where x, y, and z are defined in Fig. C and the subscripts 1 and 2 indicate the in-plane and out-of-plane components, respectively. As is conventionally done, by assuming the plane stress state ($\sigma_{zz} = \sigma_{yz} = \sigma_{zx} = 0$) [S4], we have

$$\begin{pmatrix} \epsilon_{xx} \\ \epsilon_{yy} \\ 2\epsilon_{xy} \end{pmatrix} = \begin{pmatrix} 1/E_1 & -\nu_1/E_1 & 0 \\ -\nu_1/E_1 & 1/E_1 & 0 \\ 0 & 0 & \frac{2(1+\nu_1)}{E_1} \end{pmatrix} \begin{pmatrix} \sigma_{xx} \\ \sigma_{yy} \\ \sigma_{xy} \end{pmatrix}. \tag{S24}$$

Thus, only the in-plane components appear under the condition. Note that the in-plane bulk and shear moduli are defined as $h(\sigma_{xx}+\sigma_{yy})/2 = K_{\text{in-plane}}(\epsilon_{xx}+\epsilon_{yy})$ and $h\sigma_{xy} = 2G_{\text{in-plane}}\epsilon_{xy}$, where h is the height of cell sheet. Therefore, we obtain

$$K_{\text{in-plane}}/h = \frac{E_1}{2(1-\nu_1)} \tag{S25}$$

$$G_{\text{in-plane}}/h = \frac{E_1}{2(1+\nu_1)}. \tag{S26}$$

These equations allow us to translate the in-plane moduli into the three-dimensional moduli.

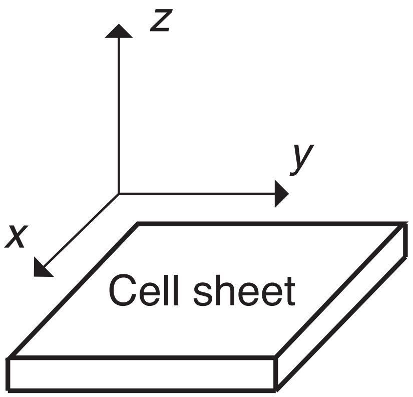

FIG. C. Definition of x, y, and z axes.

[S1] Nier, V. *et al.* Inference of internal stress in a cell monolayer. *Biophysical Journal* **110**, 1625–1635 (2016).
[S2] Rauch, H. E., Striebel, C. T. & Tung, F. Maximum likelihood estimates of linear dynamic systems. *AIAA Journal* **3**, 1445–1450 (1965).
[S3] Marmottant, P. *et al.* The role of fluctuations and stress on the effective viscosity of cell aggregates. *Proceedings of the National Academy of Sciences* **106**, 17271–17275 (2009).
[S4] Tambe, D. T. *et al.* Monolayer stress microscopy: Limitations, artifacts, and accuracy of recovered intercellular stresses. *PLoS ONE* **8**, 1–12 (2013).
